# Identification of a potent MAR element from the human genome and assessment of its activity in stably transfected CHO cells

**DOI:** 10.1111/jcmm.13361

**Published:** 2017-10-27

**Authors:** Zheng‐Wei Tian, Dan‐Hua Xu, Tian‐Yun Wang, Xiao‐Yin Wang, Hong‐Yan Xu, Chun‐Peng Zhao, Guang‐Hua Xu

**Affiliations:** ^1^ Department of Biochemistry and Molecular Biology Xinxiang Medical University Xinxiang Henan China

**Keywords:** matrix attachment region, Chinese hamster ovary cell, stable transgene expression

## Abstract

Low‐level and unstable transgene expression are common issues using the CHO cell expression system. Matrix attachment regions (MARs) enhance transgene expression levels, but additional research is needed to improve their function and to determine their mechanism of action. MAR‐6 from CHO chromosomes actively mediates high and consistent gene expression. In this study, we compared the effects of two new MARs and MAR‐6 on transgene expression in recombinant CHO cells and found one potent MAR element that can significantly increase transgene expression. Two MARs, including the human CSP‐B MAR element and *DHFR* intron MAR element from CHO cells, were cloned and inserted downstream of the poly(A) site in a eukaryotic vector. The constructs were transfected into CHO cells, and the expression levels and stability of *eGFP* were detected by flow cytometry. The three MAR sequences can be ranked in terms of overall *eGFP* expression, in decreasing order, as follows: human CSP‐B, *DHFR* intron MAR element and MAR‐6. Additionally, as expected, the three MAR‐containing vectors showed higher transfection efficiencies and transient transgene expression in comparison with those of the non‐MAR‐containing vector. Bioinformatics analysis indicated that the NFAT and VIBP elements within MAR sequences may contribute to the enhancement of *eGFP* expression. In conclusion, the human CSP‐B MAR element can improve transgene expression and its effects may be related to the NFAT and VIBP elements.

## Introduction

The demand for recombinant therapeutic proteins has grown substantially, and many proteins can only be produced in mammalian cells owing to the capacity for post‐translational modification and human protein‐like molecular structure assembly 1. Nearly 70% of currently approved recombinant proteins are generated in Chinese hamster ovary (CHO) cells, which are the preferred choice for recombinant glycoprotein production 2, 3. CHO cells have the capacity for accurate post‐transcriptional modification and proteins produced by these cells are similar to the natural molecules with respect to molecular structure, physical and chemical properties, and biological functions 4, 5. With the development and application of serum‐free culture technology, genetic engineering and large‐scale culture technologies, the CHO system is widely used in research and for the production of antibodies, recombinant proteins and vaccines 6.

Low recombinant protein expression levels and transgene silencing are common issues in current recombinant protein production and can be caused by positional effects related to neighbouring chromatin 7, 8. To overcome these issues, gene regulatory elements, such as insulators, ubiquitous chromatin opening elements, expression augmenting sequence elements, stabilizing and anti‐repressor elements, and MARs 9, 10, 11, are used to increase recombinant protein production.

Previous studies have demonstrated that MARs could enhance transgene expression, decrease variation among transformants in the process of gene expression 12, 13, 14, 15, 16 and overcome gene inactivation. MARs are genomic DNA regions that can facilitate the anchoring of the chromatin structure to the nuclear matrix 17. In addition, it has been suggested that MAR activity is not related to the DNA itself, but to structural conformations formed by the DNA. The features of MARs include AT‐rich DNA topoisomerase‐binding sites, origins of replication, special AT‐rich binding protein (STAB) motifs, kinked DNA and curved DNA 18.

Several MARs increase protein production in mammalian cells, including human β‐globin MARs 19, MARs from CHO cell chromosomes and MARs from chicken genomic DNA. However, improvements in function and analyses of the underlying mechanism are necessary. In this study, we characterized a new and more powerful MAR element from the human genome; this MAR can be used to improve transgene expression in transfected CHO cells.

## Materials and methods

### Plasmids and constructs

**Figure 1:**
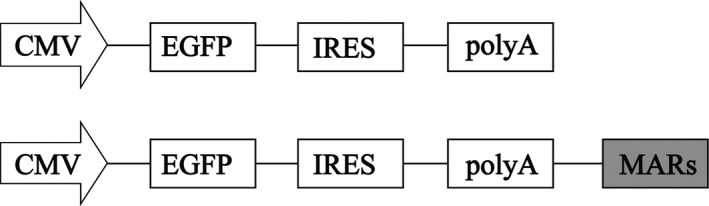
Representation of the constructs containing different MARs. Schematic illustration of expression vector containing different MARs downstream of Poly A. CMV, human cytomegalovirus IE gene promoter; *eGFP*, enhanced green fluorescence protein; IRES, internal ribosome entry site; poly A, polyadenylic acid.

Human CSP‐B MAR (hu‐MAR) (GenBank No. M62716), MAR‐6 from CHO cells (MAR1) 20 (GenBank No. NW_003613799.1, position 724006–725949) and MAR from the *DHFR* intron (MAR2) 21 (GenBank No. X06654) were artificially synthesized by General Biosystems (Chuzhou, China) based on the reported sequence. They were cloned into the region downstream of the poly(A) sequence in the pIRES‐EGFP vector, which was obtained by cloning the enhanced green fluorescent protein (*eGFP*) from pEGFP‐C1 (Clontech, Mountain View, CA, USA) into the pIRES‐neo vector (Clontech), thereby producing pIRES‐MAR, pIRES‐CSP and pIRES‐DHFR vectors. The resultant vectors were confirmed by restriction enzyme digestion and sequencing (Fig. 1).

### Cell culture and transfection

CHO‐S cells (#A11557‐01; Life Technologies, Carlsbad, CA, USA) were cultured in serum‐free medium in a humidified incubator for suspension culture at 37°C with 5% CO_2_. The cells were plated at a density of 1.5 × 10^5^ cells/well in 24‐well plates. After about 24 hrs, the cells were transfected using Lipofectamine^®^ 3000 Transfection Reagent (Thermo Fisher Scientific, Waltham, MA, USA) according to the manufacturer's instructions. About 48 hrs after transfection, CHO cells were collected and cultured in a culture medium supplemented with 800 μg/ml G418 (Invitrogen, Waltham, MA, USA) for 2 weeks. Subsequently, cell populations exhibiting stable transgene integration were cultured in CD CHO medium (#10743‐029; Life Technologies) supplemented with 8 mM l‐glutamine (#25030‐024; Life Technologies) in 125‐ml Corning shake flasks (#431255; Sigma‐Aldrich, St. Louis, MO, USA) with 30 ml of medium in the presence of 500 μg/ml G418 for 10–15 days; at 70–80% density, cells were collected for analysis.

### Flow cytometry

To determine *eGFP* expression levels, cells (4 × 10^5^ cells/ml) were seeded in 6‐well plates. At 10 generations after adding the G418 supplement, *eGFP* expression levels in cells were analysed using a FACSCalibur cytometer (Becton Dickinson, Franklin Lakes, NJ, USA). A total of 100,000 fluorescent events were acquired using a 530/15 bandpass filter for the green fluorescent protein signal acquired with a fluorescence emission wavelength of 530 nm.

### Stability testing

After transgene expression was detected by flow cytometry at generation 10 with the G418 supplement, the stably screened CHO cells transfected with hu‐MAR, MAR1 and MAR2 were further cultured in medium supplemented with G418 until 40 generations. Then, the expression of *eGFP* was analysed using a FACSCalibur instrument (Becton Dickinson) again to analyse recombinant gene expression stability.

### Fluorescence quantitative PCR

Relative *eGFP* gene copy numbers were measured by fluorescence quantitative PCR (qPCR). Genomic DNA was extracted from stable cells according to the manufacturer's instructions (TaKaRa, Dalian, China). The primers used for the fluorescence qPCR were as follows: *eGFP*, F1, 5′‐CTACGTCCAGGAGCGCACCATCT‐3′ and R1, 5′‐GTTCTTCTGCTTGTCGGCCATGATAT‐3′. The glyceraldehyde phosphate dehydrogenase (*GAPDH*) gene was used as an internal reference, and the primer sequences were as follows: F1, 5′‐CGACCCCTTCATTGACCTC‐3′ and R1, 5′‐CTCCACGACATACTCAGCACC‐3′. qPCRs were performed using the ABI 7500 SYBR Fluorescence quantitative PCR instrument (Applied Biosystems, Foster City, CA, USA), and 7500 Fast System SDS Software was used to analyse the results. The cycling parameters were as follows: 95°C for 3 min.; and 35 cycles of 94°C for 30 sec., 50°C for 30 sec. and 72°C for 30 sec. The qPCRs were performed using the Platinum SYBR Green qPCR SuperMix‐UDG Kit (Invitrogen). The 2^−ΔΔCt^ method was used to calculate the relative *eGFP* copy numbers.

### Bioinformatics analyses

Bioinformatics analyses were performed according to the methods described in a previous study 8. MatInspector (http://www.genom atix.de/products/index.html) was used to analyse allele‐specific transcription factor binding sites. Structural motifs were identified using GeneExpress.

### Statistical analysis

All experimental data were analysed using SPSS 18.0 (SPSS Inc., Chicago, IL, USA). Data are reported as means ± standard deviation. Comparisons among groups were analysed using a single‐factor analysis of variance, and *t*‐tests were used for pairwise comparisons. *P* < 0.05 was considered statistically significant.

## Results

### Transfection efficiency and transient expression of recombinant protein

**Figure 2:**
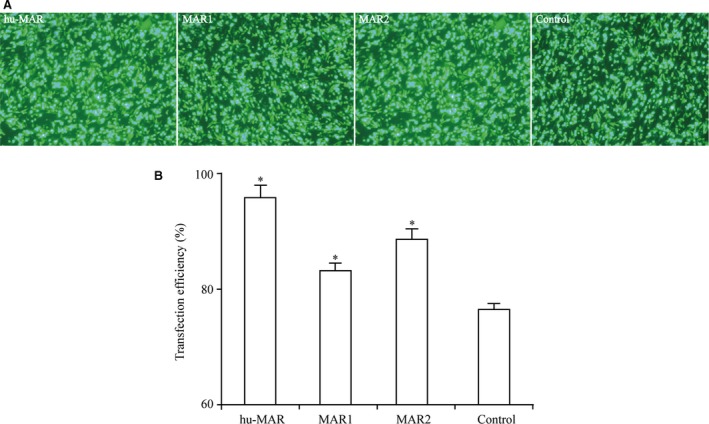
Transfection efficiency of different MARs in transfected pools. The four constructed vectors were transfected into CHO‐S cells using Lipofectamine^®^ 3000 Transfection Reagent (Thermo Fisher Scientific) according to the manufacturer's instructions. The transfection efficiencies and *eGFP* expression were estimated using an epifluorescence microscope after 48 hrs of transfection. (**A**) Transfection efficiencies observed by fluorescence microscopy. (**B**) Analysis of the transfection efficiency. Three independent experiments were performed in this study. Standard error of the mean (S.E.M.) is indicated (Student's *t*‐test, *P < 0.05). hu‐MAR: human CSP‐B MAR; MAR1: MAR‐6 from CHO cells, MAR2: MAR from the *DHFR* intron.

We first evaluated the transfection efficiency of hu‐MAR, MAR1 and MAR2 in CHO cells. All three MARs had higher transfection efficiencies than that of the control vector (Fig. 2A and B). Additionally, the transfection efficiencies differed among the three MARs; it was highest for hu‐MAR at approximately 96%, followed by MAR2 at approximately 89% and MAR1 at approximately 83%. Transfection efficiency may be related to the structure and length of MARs; hu‐MAR was the second longest, but showed the highest transfection efficiency.

**Figure 3:**
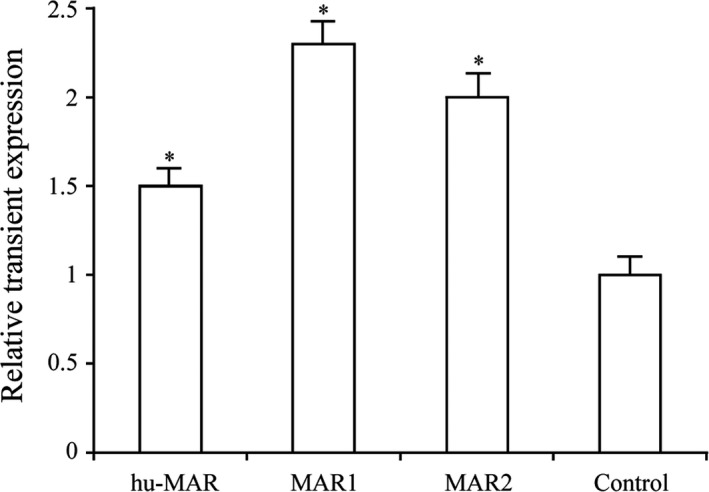
Transient expression of recombinant protein of different MARs in transfected pools. The constructed vectors were transfected into CHO‐S cells, and *eGFP* expression levels were estimated by epifluorescence microscopy after 48 hrs of transfection. Three independent experiments were performed in this study. Standard error of the mean (S.E.M.) is indicated (Student's *t*‐test, *P < 0.05).

We also observed that hu‐MAR and MAR2 resulted in significantly higher transient *eGFP* expression than that of the control vector. The enhancement was highest for hu‐MAR, which improved transgenic *eGFP* expression by 2.3‐fold, followed by the MAR2 (2.0‐fold). MAR1 resulted in a slight increase in transgene expression after transient transfection (Fig. 3).

### Recombinant protein expression in stably transfected cells

**Figure 4:**
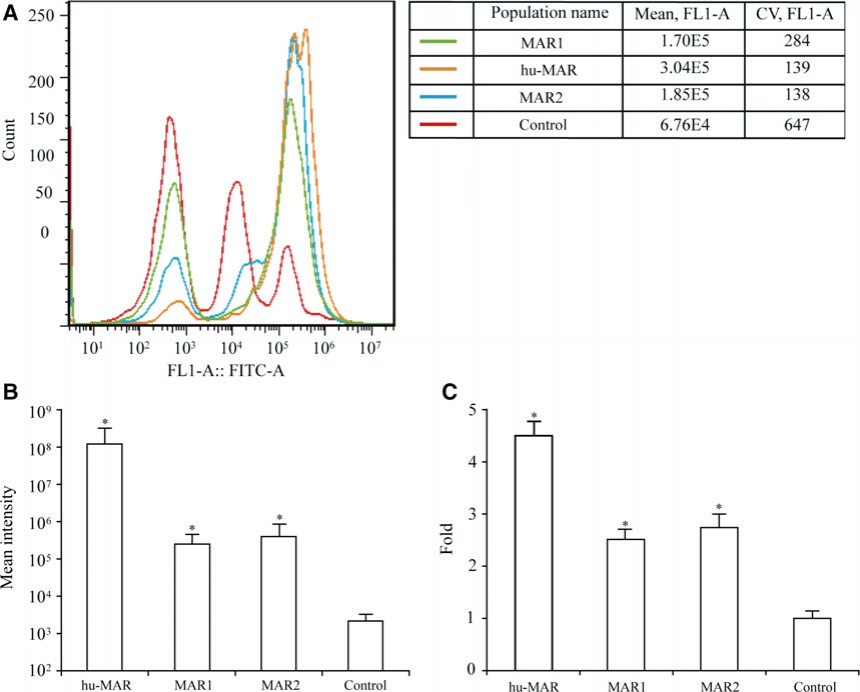
MARs increased transgene expression in stable transfected pools. The *eGFP* MFI was measured after culturing cells 35 days with G418 supplement. *eGFP* expression levels in the cells were directly analysed using a FACSCalibur cytometer (Becton Dickinson) (**A, B**). *eGFP* MFI expression was normalized to a control vector lacking MAR, and in the statistical analysis of *eGFP* expression, fold change values were normalized to those of the control vector without MAR (**C**). Three independent experiments were performed in this study. Standard error of the mean (S.E.M.) is indicated (Student's *t*‐test, *P < 0.05).

CHO cells were transfected with vectors and subjected to drug selection in order to establish stable transfectants. We cultured colonies of stably transfected CHO cells with drugs agents for 10 generations. The expression of *eGFP* was measured by flow cytometry in stable transfectants. The median fluorescence intensity was higher in stably transfected cell lines with MARs than in cells transfected with the control vector (Fig. 3A and B). MARs could improve *eGPF* expression in stably transfected pools. The hu‐MAR vector resulted in the highest *eGFP* expression, that is 4.50‐fold higher than that for the vector without MARs. MAR2 and MAR1 increased *eGFP* expression by approximately 2.74‐ and 2.51‐fold, respectively (Fig. 4C).

### Analysis of long‐term recombinant protein expression stability

**Figure 5:**
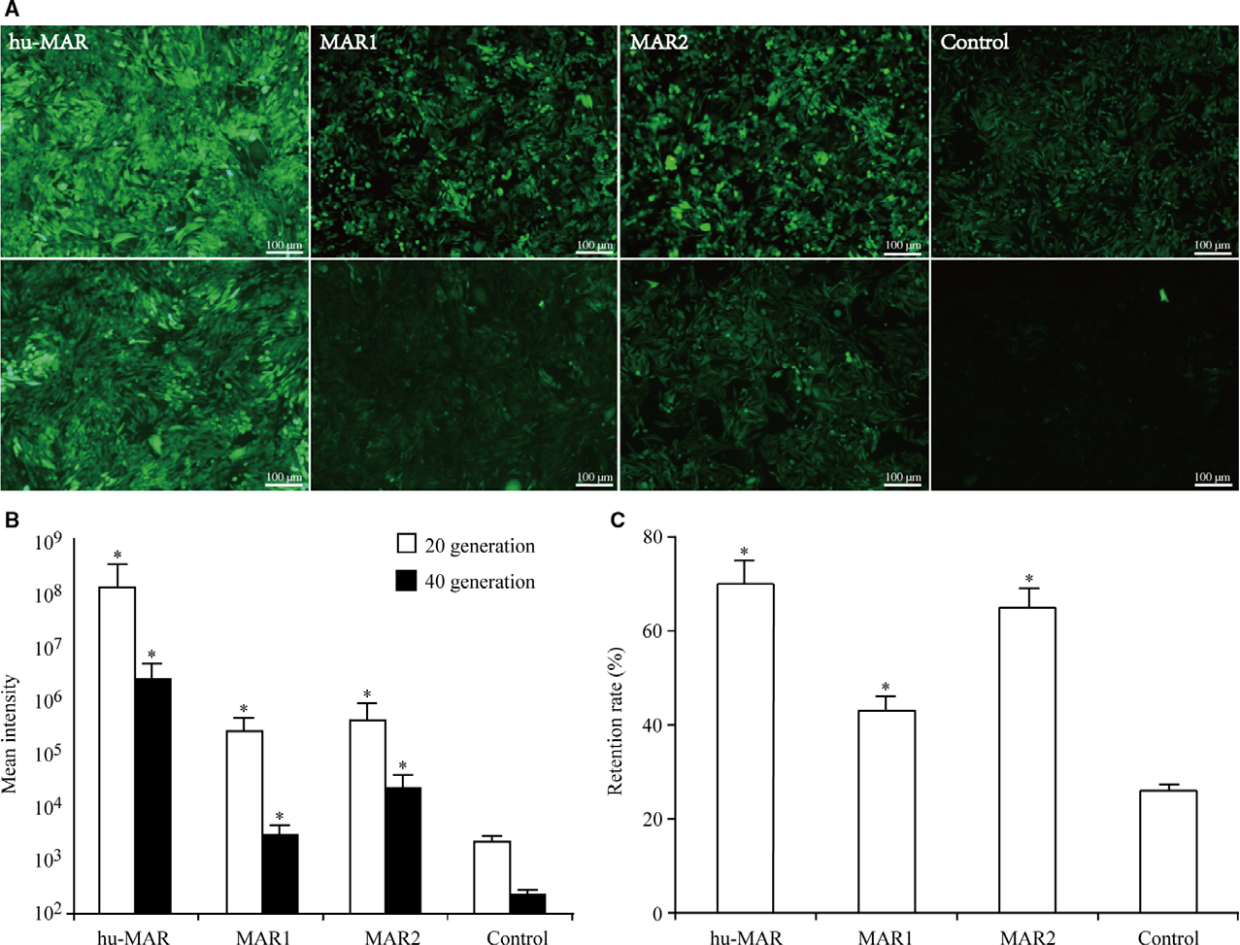
The levels and retention of *eGFP* in stable transfected CHO cells. The stably transfected CHO cells were cultured into a culture medium supplemented with 400 μg/ml G418 (Invitrogen) for 20 generations and for 40 generations, and the stability of transgene expression was detected by fluorescence microscopy and flow cytometry. (**A**) Fluorescence microscopy results. Upper panel represents 10 generations and lower panel represents 20 generations. (**B**). Relative changes in *eGFP* expression levels in cells at different days post‐transfection were directly analysed using a FACSCalibur cytometer (Becton Dickinson, Franklin Lakes, NJ, USA). (**C**) The *eGFP* expression retention was calculated as the ratio of the MFI at the end of stability testing to the MFI at 35 days of stability testing. Three independent experiments were performed in this study. Standard error of the mean (S.E.M.) is indicated (Student's *t*‐test, *P < 0.05).

To analyse the effect of MARs on long‐term recombinant protein expression stability, we evaluated *eGFP* expression in CHO cells cultured for 40 generations by measuring the median fluorescence intensity for each stable cell pool. Based on fluorescence microscopy and flow cytometry analyses, *eGFP* expression decreased in cells transfected with hu‐MAR, MAR1 and MAR2 to some extent and was significantly lower than the expression in cells transfected with vectors lacking MARs (Fig. 5A and B). The retention rates of hu‐MAR, MAR2 and MAR1 were 70%, 65% and 43%, respectively (Fig. 5C). However, the retention rate of *eGFP* was only 26% for the control vectors. These results suggested that MAR has a role in strengthening and maintaining *eGFP* expression, and hu‐MAR was most effective with respect to maintaining transgene expression. These results suggested that MARs could have a positive effect on recombinant protein expression stability.

### Gene copy number analysis

**Figure 6:**
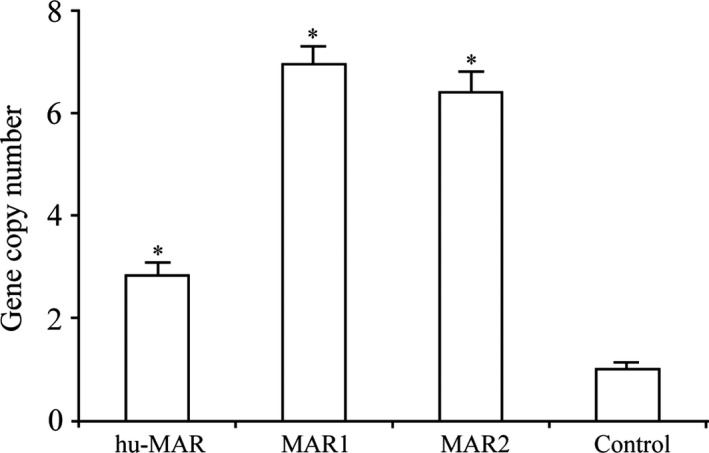
Relative *eGFP* gene copy numbers in stable transfected cells. We used fluorescent quantitative PCR to measure relative *eGFP* gene copy numbers. The 2^−ΔΔCt^ method was used to calculate relative *eGFP* copy numbers. The *eGFP* gene copy numbers were normalized to the control vector whose value was set to 1. Three independent experiments were performed in this study. Standard error of the mean (S.E.M.) is indicated (Student's *t*‐test, *P < 0.05).

To investigate the correlation between *eGFP* expression levels and *eGFP* gene copy number, we performed fluorescence qPCR using genomic DNA extracted from stably transfected CHO cells. As shown in Figure 6, gene copy numbers in cells transfected with the vector containing MARs were all higher than the copy number for cells transfected with the control vector. However, we did not detect a linear relationship between gene copy number and transgene expression level. The mean gene copy number was lower for cells transfected with hu‐MAR than for cells transfected with MAR1 and MAR2 (2.80 ± 0.43, 6.90 ± 1.21 and 6.40 ± 0.89, respectively), but the *eGFP* expression levels were highest for cells transfected with hu‐MAR. These results suggested that the levels of *eGFP* expression were not only related to gene copy number, but were determined by other mechanisms.

### Bioinformatics analyses

According to a previous report, transcription factor binding motifs, such as FAST‐1, SATB1 and C/EBP binding sites, might improve transgene expression. In this study, three MARs were analysed using a bioinformatics approach. The results indicated that NFAT and VIBP elements within MAR sequences may contribute to the enhancement of *eGFP* expression, suggesting that the positions of certain transcription factor binding sites contribute to the increase in transgene expression (Table 1).

**Table 1:** Locations of various transcription factor binding motifs within the three MARs

Matrix Family	CSP‐B	DHFR	MAR‐6	Strand
CEBP	1	0	3	+
	3	0	7	−
MYT1	4	1	6	+
	7	2	6	−
FAST	3	1	1	+
	4	2	6	−
GATA	2	1	4	+
	4	1	2	−
NFAT	3	0	3	+
	4	3	3	−
VTBP	5	6	2	+
	6	2	5	−

Transcription factor binding motifs, such as FAST‐1, SATB1 and C/EBP binding sites might contribute to improve transgene expression. In this study, bioinformatic analyses detected CEBP, MYT1, FAST, GATA, NFAT and VTBP transcription factor binding sites within the three MARs. NFAT and VIBP elements may contribute to the enhancement of EGFP expression mediated by the MAR sequence. These results suggest that the positioning of certain transcription factor binding sites contributed to transgene expression.

## Discussion

MARs have been studied extensively to determine their roles in gene expression, chromatin organization and DNA replication. Previous studies have suggested that MAR elements could enable more effective and more stable gene expression in CHO cells 22, 23, 24. MAR1 from CHO cells improves the level and stability of gene expression in CHO‐K1 cells, and these effects are stronger than those of the chicken lysozyme MAR.

In the present study, we compared the effects of hu‐MAR and MAR2 with those of MAR1 on transgene expression in CHO cells using the *eGFP* reporter gene. *eGFP* is frequently used as a reporter to investigate the regulatory effect of cis‐acting elements. In previous studies, the *eGFP* gene was initially used to study the effect of MAR on transgene expression in transfected CHO cells, and genes of interest (GOI), such as antibodies and other proteins, were further studied. GOI expression levels were consistent with the levels of *eGFP* 12, 14, 25. We will investigate some the production of antibodies and other biopharmaceutical proteins in future studies.

We found that the three MARs could increase the transfection efficiency as compared to that of the control vector. The size of the vector influenced transfection efficiency to some extent, but the structure and configuration of MARs had significant effects on transfection efficiency. In the present study, MAR1 was the longest, followed by hu‐MAR and MAR2. However, the hu‐MAR showed the highest transfection efficiency, followed by MAR2. The transfection efficiency in CHO cells was higher for MAR‐containing constructs than for the control vector, consistent with the results of a previous study 26.

Hu‐MAR improved the transient and stable expression of recombinant protein in transfected CHO cells compared with that of the control. In general, MARs are not considered to improve transient transfections; it is possible that transgene genomic integration and/or changes in chromatin structure are not obvious in transient transfections. However, a few studies have demonstrated that MARs increase transgene expression in transient transfections 27, 28. There are various mechanisms by which MARs increase transgene expression after stable transfections; MAR may prevent the spread of heterochromatin, thereby preventing transgene silencing, or it may recruit chromatin remodelling proteins. In the present study, all three tested MARs enhanced transgene expression levels in stably transfected CHO cells.

MARs show different activities due to specific DNA motifs. Previous studies have shown that the AT cores may bind to some special transcription factors that target AT‐rich sequences, as suggested by earlier bioinformatics modelling studies 25, 29, 30, 31. The results confirmed that AT‐rich sequences are essential for MAR‐mediated increases in transgene expression 32 and hu‐MAR had the highest AT content (approximately 69%). The DNA base pairs may be key features for MAR activity in terms of increased transgene expression 33. Curved DNA motifs are good indicators of active MAR portions, and binding sites for transcription factors (*e.g*. SatB1, NMP4 and Hox‐like family proteins) are vital for MAR activity. In this study, bioinformatics analyses indicated that NFAT and VIBP elements within MAR sequences may contribute to the enhancement of *eGFP* expression.

In addition, MAR1 has been reported to confer high activity, but was not the most effective MAR in our study. We suspect that the backbone, cell type and source of MARs may determine their effect on transgene expression. According to a previous study, elements of the backbone vector influence MAR activity with respect to increasing transgene expression 34.

Previous studies have demonstrated that MARs increase transgene expression and improve the stability of transfected cells as compared to cells transfected with a control vector lacking MARs. In the present study, all three MARs functioned to increase transfection stability, consistent with the results of a previous study 21, 35.

It has been reported that MAR elements enhance transgene expression by facilitating copy number‐dependent or position‐independent expression 36, 37, 38, 39. However, some reports have indicated that the activity of MARs is not sufficient to confer copy number‐dependent transgene expression in animal systems 40. Our results showed that MARs can improve transgene copy number, but gene copy number and transgene expression were not linearly related, indicating that the levels of *eGFP* expression were not only related to gene copy number, but to other mechanisms.

In conclusion, in the present study, we first identified a potent MAR element (human CSP‐B MAR) able to improve transgene expression in both transient and stable transfections. The effects may not be directly related to the increase in transgene copy numbers; other factors are also likely to affect transgene expression. Additional studies are required to optimize other cis‐acting elements, investigate the GOI for recombinant protein production and further elucidate the mechanisms underlying these effects.

## Conflict of interest

All authors have no conflict of interest regarding this article.
